# Study on Ti-6Al-4V Alloy Machining Applying the Non-Resonant Three-Dimensional Elliptical Vibration Cutting

**DOI:** 10.3390/mi8100306

**Published:** 2017-10-13

**Authors:** Mingming Lu, Jiakang Zhou, Jieqiong Lin, Yan Gu, Jinguo Han, Dongpo Zhao

**Affiliations:** Key Laboratory of Micro/Nano and Ultra-Precision Manufacturing (Jilin Province), School of Mechatraonic Engineering, Changchun University of Technology, Changchun 130012, China; lumm@ccut.edu.cn (M.L.); zhoujiakang@ccut.edu.cn (J.Z.); guyan@ccut.edu.cn (Y.G.); hankeyee@ccut.edu.cn (J.H.); zhaodongpo@ccut.edu.cn (D.Z.)

**Keywords:** Ti-6Al-4V alloy, non-resonant, three-dimensional elliptical vibration cutting (3D-EVC), topography, freeform surface

## Abstract

The poor machinability of Ti-6Al-4V alloy makes it hard to process by conventional processing methods even though it has been widely used in military and civilian enterprise fields. Non-resonant three-dimensional elliptical vibration cutting (3D-EVC) is a novel cutting technique which is a significant development potential for difficult-to-cut materials. However, few studies have been conducted on processing the Ti-6Al-4V alloy using the non-resonant 3D-EVC technique, the effect of surface quality, roughness, topography and freeform surface has not been clearly researched yet. Therefore, the machinability of Ti-6Al-4V alloy using the non-resonant 3D-EVC apparatus is studied in this paper. Firstly, the principle of non-resonant 3D-EVC technique and the model of cutter motion are introduced. Then the tool path is synthesized. The comparison experiments are carried out with traditional continuous cutting (TCC), two-dimension elliptical vibration cutting (2D-EVC), and the non-resonant 3D-EVC method. The experimental results shown that the excellent surface and lower roughness (77.3 nm) could be obtained using the non-resonant 3D-EVC method; the shape and dimension of elliptical cutting mark also relates to the cutting speed and vibration frequency, and the concave/convex spherical surface topography are achieved by non-resonant 3D-EVC in the Ti-6Al-4V alloy. This proved that the non-resonant 3D-EVC technique has the better machinability compared with the TCC and 2D-EVC methods.

## 1. Introduction

Titanium alloy, which enjoyed a reputation as the metal of the future, was widely used in the shipbuilding, chemical, electronics and communication equipment fields owing to its unique mechanical properties such as high strength, high heat resistance, excellent corrosion resistance, and so on. It is an indispensable material for rockets, missiles and space shuttles [1,2]. However, the poor processing property and machinability of titanium alloy made it hard to obtain the ideal machining results by conventional processing methods. The reason was that the contact length between chip and tool rake face was extremely short, and the generated heat was not easy to spread due to its high heat capacity and low thermal conductivity. Moreover, the cutting heat mainly formed in narrow region adjacent to cutting zone and cutting edge, and the highest temperature value in this region that could be reached was 1000 °C [3,4]. Simultaneously, the chipping phenomenon would appear with greatly increasing cutting force acting on the unit contact area. It was easy to cause deformation under the action of radial cutting force due to the low elastic modulus of titanium alloy: it would produce the vibration, increase tool wear and affect the surface quality [5,6]. In addition, it also led to rapid hardening due to the absorption of oxygen and nitrogen at higher cutting temperatures caused by the high chemical reactivity and the severe plastic deformation of titanium alloy [7].

Therefore, many researchers have studied the cutting methods of titanium alloy in order to obtain better cutting performance and increase the machining efficiency in recent years. This has included the material choice of cutting tools which have a high degree of hardness and great cutting performance, high-pressure coolant was used to relieve the adhesive and diffusive effect, and improvements to the cutting method to increase the cutting property. Some tools with excellent cutting performance, such as polycrystalline cubic boron nitride (PCBN) tools and polycrystalline diamond (PCD) tools, have recently been used in the machining of difficult-to-cut material. PCBN tools could achieve steady cutting at high temperatures and get a higher surface processing quality with high hardness, abrasion resistance and chemical stability. However, the PCBN tools also wear rapidly owing to the adhesive wear and diffusive caused by the high chemical reactivity in the continuous cutting process of titanium alloy [7,8]. PCD tools have the characteristics of high hardness, high compressive strength, good thermal conductivity and good wear resistance. The friction coefficient of PCD tools is generally only 0.1~0.3 (the friction coefficient of cemented carbide is 0.4~1), which can significantly reduce cutting force in machining process [9,10]. However, they also experience adhesive wear easily under the conditions of high cutting temperature and large cutting force in the unit area during the processing of titanium alloy. The high-pressure coolant could be employed to relieve adhesive wear and prolong the tool life effectively, but it was impossible to reduce the heat concentrating in the narrow region adjacent to the cutting zone and cutting edge [11].

Recently, some researches demonstrated that the processing property and machinability of titanium alloy could be improved during the process of intermittent cutting, and it performed better than the traditional continuous cutting (TCC) in cutting force, temperature and chip removal rate [12,13]. Typical intermittent cutting methods was developed based on vibration cutting. Recently, the vibration assisted cutting technology has been widely used in polishing of difficult-to-cut materials [14,15], wire electrochemical micro machining [16], milling [17,18] and other processing fields. The research shows that the vibration assisted cutting technology can obviously improve cutting performance of difficult-to-machine materials, and the specific performance of its great processability in the following: surface removal rate; cutting force; machining efficiency, and; machining quality. In the cutting process, each cutting cycle was composed of the effective cutting time and the non-contact movement time during intermittent cutting process. In other words, tool and workpiece remained contact intermittently and periodically, hence the tool obtained a periodic break in each cutting cycle [19]. Ultrasonic vibration machining is a typical method of intermittent cutting. Sui et al. proposed a high-speed ultrasonic vibration cutting (UVC) method and applied to the cutting process of titanium alloy, and it obtained a great improvement in tool life, processing efficiency, cutting force and surface quality compared with TCC [20]. However, the instantaneous cutting force in process of high-speed UVC was much larger, and the relationship between cutting depth and separation condition of tool-workpiece was difficult to control. In addition, the vibration assisted cutting technology also could produce the desired workpiece surface topography. For example, Zhu developed a novel 2D vibration-assisted compliant cutting system to generate various complex textured surfaces uniformly and accurately, the evaluation results shown that the complex textured surfaces was matched with the theoretically predicted surfaces in in-process monitored parameters [21]. Another typical intermittent cutting method is the two-dimension elliptical vibration cutting (2D-EVC) proposed by Shamoto in 1994 [22]. The cutting edge formed an elliptical locus by synchronizing 2D vibration in the plane, including the cutting direction and chip flow direction. Further studies indicated that the cutting force and cutting temperature with elliptical vibration were much lower than those without vibration due to the effect of intermittent cutting and intermittent separation of the tool-workpiece [23]. The cutting performance of difficult-to-cut material, such as the Inconel 718 and sintered tungsten carbide, were also studied by combining the 2D-EVC apparatus, the cutting experiment results shown that the cutting force, cutting temperature, surface quality and the effects of cutting-tool wear with the 2D-EVC method were much smaller than without vibration cutting method [24,25].

In order to expand the processing requirements of different surfaces and overcome the limitations of two-dimensional elliptical trajectory machining surfaces, the resonance three-dimensional elliptical cutting (3D-EVC) was proposed in 2005 with better chip removal, lower cutting force and wider surface machining [26,27,28]. The elliptical trajectory of this resonance apparatus was generated by synthetizing two transverse vibrations and a longitudinal vibration based on the cutter bar. The unique characteristics of this resonance 3D-EVC apparatus were that the plane orientation of elliptical motion generated in tool tip point could be adjusted to locate the plane of chip flow. However, the parameters of elliptical motion trajectory (e.g., frequency and amplitude of vibration) in existing resonant 3D-EVC apparatus were difficult to adjust, and could not adapt to the processing requirements of different materials and the surface. Therefore, the non-resonant 3D-EVC apparatus was developing gradually in order to overcome the limitations of the resonance 3D-EVC apparatus. At present, the research on non-resonant 3D-EVC technology is mainly focused on device design and optimization, path planning, cutting force modeling and system identification [29,30,31]. However, non-resonant 3D-EVC has been used in sculptured surface machining of die steel, and the mirror surfaces of hardened steel are obtained successfully [32]. However, few studies have been conducted on processing of titanium alloy using the non-resonant 3D-EVC technique. Moreover, compared with TCC and 2D-EVC methods, the effect of surface quality, roughness and complex surface quality obtained by using the non-resonant 3D-EVC method in titanium alloy have not been clearly researched yet.

In this paper, a new non-resonant 3D-EVC apparatus is proposed to examine whether the non-resonant 3D-EVC technique is beneficial during the processing of Ti-6Al-4V alloy. Firstly, the principle of non-resonant 3D-EVC and the model of cutter motion are introduced in Section 2. Then the tool path of developed apparatus is synthesized, and the comparison experiments are carried out with traditional continuous cutting (TCC), two-dimension elliptical vibration cutting (2D-EVC) and non-resonant 3D-EVC methods in Section 3. Finally, Section 4 illustrates the results of experiments, the surface morphologies obtained by different processing methods are compared, the influence of cutting parameters (cutting speed and vibration frequency) on the cutting marks, and the machinability of the freeform surface of the Ti-6Al-4V alloy is also discussed.

## 2. Principle of Non-Resonant 3D-EVC

**Figure 1:**
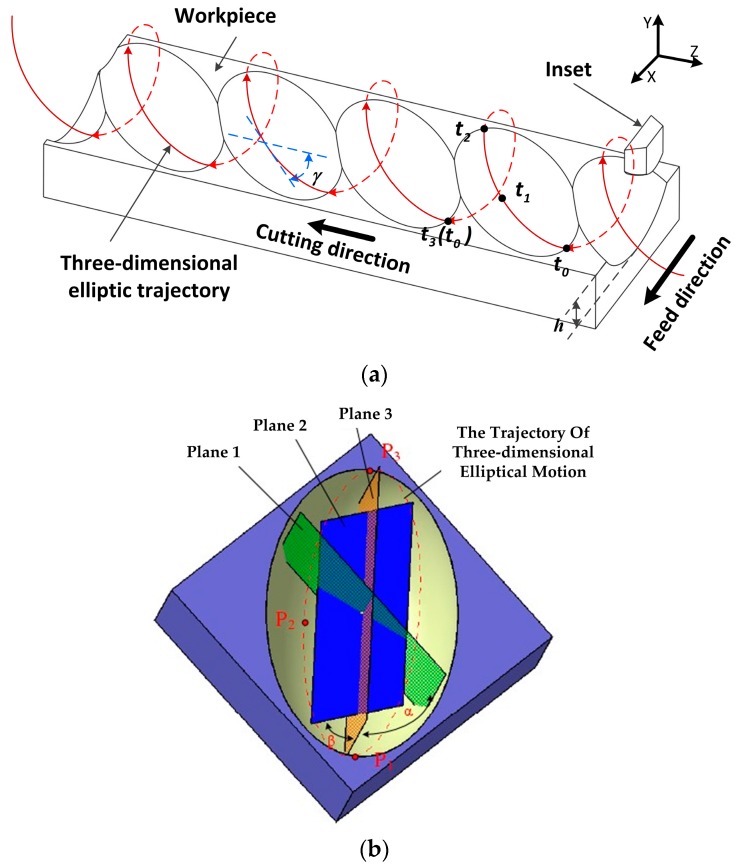
Principle of the non-resonant three-dimensional elliptical vibration cutting (3D-EVC) technique. (**a**) Cutting process of the non-resonant three-dimensional elliptical vibration cutting (3D-EVC); (**b**) Individual cutting marks in a single cutting cycle.

In non-resonant 3D-EVC, the three-dimensional elliptical motion trajectory is generated by the spatial elliptical vibration of the tool point, which can be described that certain angles of the vibration plane in 2D-EVC are offset from the workpiece along the cutting direction and depth of cut direction, respectively. The motion trajectory of tool tip in non-resonant 3D-EVC can be seen as the spatial elliptical spiral motion trajectory as shown in Figure 1. In Figure 1a, a cycle of cutting is depicted as *t*_0_-*t*_1_-*t*_2_-*t*_3_ (*t*_0_). In a cycle, the tool is separated from the workpiece in *t*_2_-*t*_3_ (*t*_0_), while it is contact with workpiece and chips in (*t*_0_) *t*_3_-*t*_1_-*t*_2_, namely, the effective cutting stage. The value *h* is the maximum depth of cut can be reached at *t*_1_. In order to obtain the intermittent cutting process and ensure the tool is separated from the workpiece and chip during each cutting vibration cycle, the cutting speed value of tool must be lower than the maximum vibration speed during non-resonant 3D-EVC process.

Figure 1b indicates the individual cutting mark in a single cutting cycle. In a single cutting mark, there are three planes to indicate the trajectory of cutting tool. Plane 1 is perpendicular to the surface of the workpiece, the plane 2 can be obtained by rotating plane 1 to α. Then, the plane 2 is rotated β to get the plane 3, which corresponds to the plane of trajectory of three-dimensional elliptical motion in the cutting process located in the tool tip. The point of *P*_1_ corresponds to the *t*_0_ time point in the effective cutting stage, which represents the point the tool begins to cut the workpiece. The point of *P*_2_ and *P*_3_ correspond to the *t*_1_ and *t*_2_ time points, which can indicate the point of maximum cutting depth that can be achieved, and the separation point of the tool and the workpiece.

In the effective cutting stage during a cutting vibration cycle, a certain angle between the movement direction of the tool on the workpiece and the cutting direction is γ, which can be adjusted by the setting of phase difference. The initial cutting and the chip forming are started at *t*_0_. The friction direction between the tool and the workpiece is reversed because of the relative movement between the workpiece and the tool is changed, which is propitious for removing the chip. The cutting force is reduced observably due to the reversal of frictional direction and the segregation phenomenon of the tool-workpiece. At the same time, the angle between the direction of the chip flow and the cutting direction of the tool can reduce the wear of the tool rake face in the non-resonant 3D-EVC process, which increases the tool life considerably.

In non-resonant 3D-EVC, the sinusoidal displacement signals input to the three piezoelectric stacks could be described as:

(1) $$\left\{ \begin{array}{l} z=A_{1}\sin(\omega_{1}t+\varphi_{1})\\ y_{1}=A_{2}\sin(\omega_{2}t+\varphi_{2})\\ y_{2}=A_{3}\sin(\omega_{3}t+\varphi_{3}) \end{array}\right.$$

where *z*, *y*_1_ and *y*_2_ are the ideal vibration displacement in *Z*, *Y*1 and *Y*2 directions of the tool tip corresponding to the coordinate system in machine tools, respectively. *A*_1_, *A*_2_ and *A*_3_ are amplitudes of driving singles in three directions generated by three piezoelectric stacks, respectively. ω_1_, ω_2_, ω_3_ and φ_1_, φ_2_, φ_3_ are the angular frequency and phase in the driving signals along *Z*, *Y*1 and *Y*2 directions, respectively. *t* is the time.

## 3. Experiments Setup

### 3.1. Apparatus of the Non-Resonant 3D-EVC

**Figure 2:**
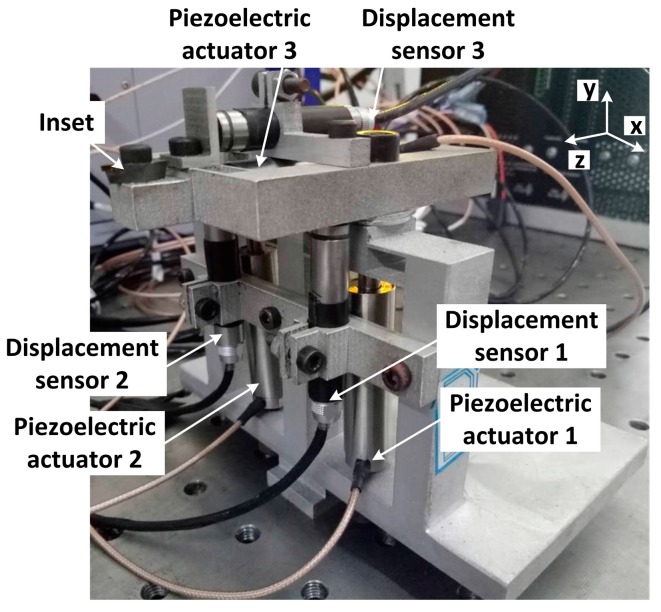
Apparatus of the non-resonant three-dimensional elliptical vibration cutting (3D-EVC) in cutting experiments.

The apparatus of the non-resonant 3D-EVC was driven by three piezoelectric actuators, as shown in Figure 2. The generation of three-dimensional elliptical motion trajectory was generated by three sinusoidal displacement signals imposed on two parallel piezoelectric actuators in the *z*-direction and a piezoelectric actuator in the *x*-direction. The three-dimensional elliptical trajectory could be controlled by adjusting the phase difference between the sinusoidal displacement signals of three piezoelectric actuators. The micro-displacement sensor is placed parallel to the piezoelectric actuator for collecting the vibration displacement of the tool point in the cutting process.

**Figure 3:**
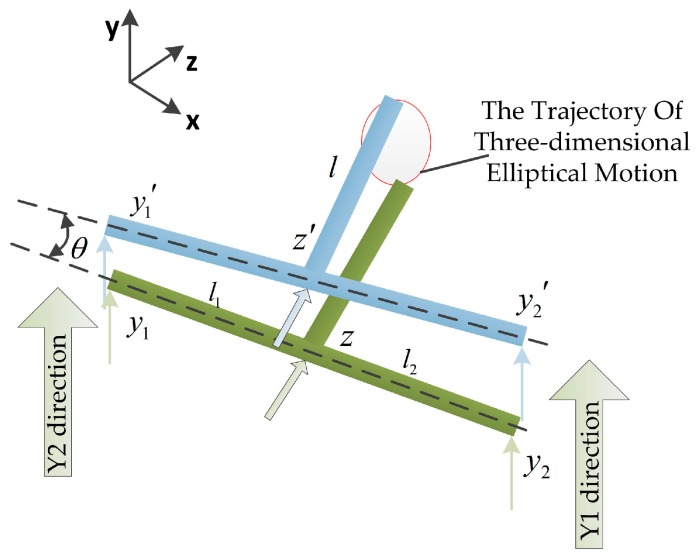
The generation principle of three-dimensional elliptical motion in the tool tip.

In order to describe the 3D elliptical trajectory of the tool tip in the process of non-resonant 3D-EVC, a model of cutter location is established. Assuming the initial position of the tool tip is *z*, *y*_1_, *y*_2_, the displacement of three piezoelectric actuators can be assumed to be *z*^′^, *y*_1_^’^, *y*_2_^′^. Therefore, the phase difference between two piezoelectric actuators installed parallel on hinge platform can be described as:

(2) $$\theta =\arcsin(\frac{y_{1}{}^{\prime }-y_{2}{}^{\prime }}{l_{1}+l_{2}})$$

where *l*_1_ and *l*_2_ represent the vertical distance from the driving point implemented on the flexible hinge along the *Y*1 and *Y*2 directions to the plane parallel to the flexible hinge along the *Z* direction of diamond tool, respectively. *l* represents the linear distance from the tool tip point to the plane of the two piezoelectric actuators in the *Y*1 and *Y*2 directions, as shown in Figure 3. In general, the three-dimensional elliptical motion trajectory of the tool tip (*x*_t_, *y*_t_, *z*_t_) in the spatial coordinate system can be represented as:

(3) $$\left\{ \begin{array}{l} x_{t}=l\times \sin\theta \\ y_{t}=l\times \cos\theta +y_{1}{}^{\prime }+y_{2}{}^{\prime }+l_{1}\times \tan\theta +l_{2}\times \tan\theta \\ z_{t}=z^{\prime } \end{array}\right.$$

**Figure 4:**
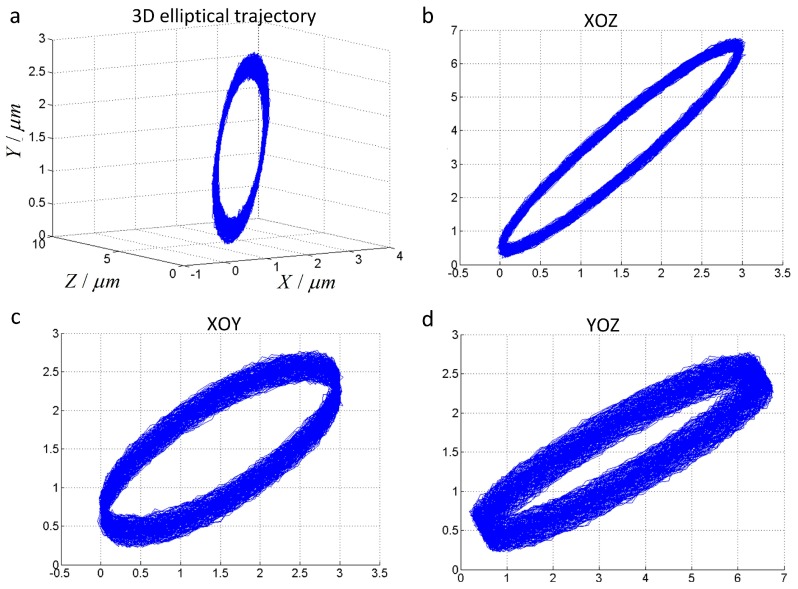
Tool trajectory of non-resonant three-dimensional elliptical vibration cutting (3D-EVC). *A*_1_ = 7 μm, *A*_2_ = *A*_3_ = 4 μm, φ_1_ = 0, φ_2_ = π/2, φ_3_ = π, ω_1_ = ω_2_ = ω_3_ = 50 Hz. (**a**) The spatial elliptical trajectory; (**b**) The projection of the 3D elliptical trajectory in the XOZ plane; (**c**) The projection of 3D elliptical trajectory in the XOY plane; (**d**) The projection of the 3D elliptical trajectory in the YOZ plane.

In this paper, the vertical distance from the driving point acted on the flexible hinge along the *Y*1 and *Y*2 directions to the plane paralleled to the flexible hinge along the *Z* direction of diamond tool are *l*_1_ = *l*_2_ = 20 mm, and the linear distance from tool tip point to the plane of the two piezoelectric actuators in *Y*1 and *Y*2 directions is *l* = 42 mm. The amplitude of driving signals executing on the piezoelectric actuators are set as *A*_1_ = 7 μm and *A*_2_ = *A*_3_ = 4 μm, respectively. The phase of three sinusoidal signals can be setting as φ_1_ = 0, φ_2_ = π/2 and φ_3_ = π, respectively. The frequency of vibration are set as ω_1_ = ω_2_ = ω_3_ = 50 Hz. Therefore, the 3D vibration displacement is synthesized by imposed the sinusoidal signals on three piezoelectric actuators, and the vibration displacement of the tool tip is measured by the probes of displacement sensor installed parallel with the piezoelectric actuators fixed by the sensor holder. The synthetic three-dimensional elliptical motion trajectory of the tool tip is shown in Figure 4.

However, there is a certain deviation between the vibration displacement of the three directions in actual synthesis and the given displacement signal. The actual values of vibration displacement are 6 μm, 3 μm and 3 μm due to the hysteresis characteristic of the piezoelectric actuators. In process of the Ti-6Al-4V alloy using the non-resonant 3D-EVC apparatus, compensating the effect of hysteresis could be achieved by adjusting the depth of cutting. In addition, the poor smoothness and burrs will be appeared in actual synthesis of 3D elliptical motion trajectory due to the machining environmental noise, control accuracy and tracking error. The effect of poor smoothness and burrs could be neglected due to the deviation is small in the actual machining process.

### 3.2. Experiments Conditions

**Figure 5:**
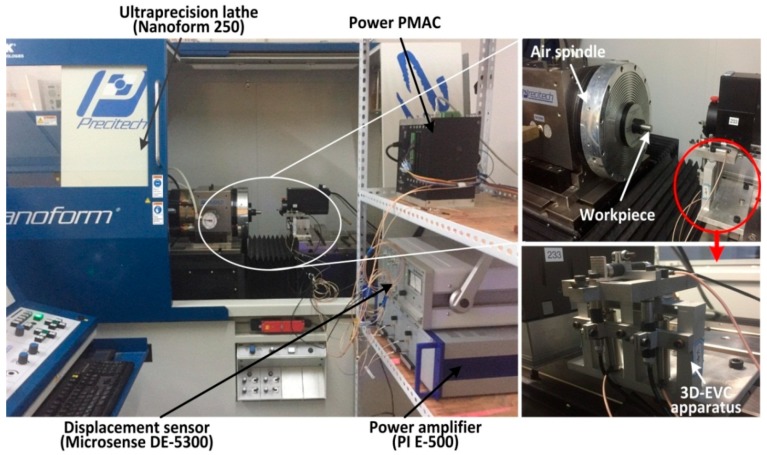
Machine setup of cutting experiments.

The cutting experiments of Ti-6A1-4V alloy were carried out on the ultra-precision machine tools (Nanoform 250, AMETEK Precitech Inc., Keene, NH, USA), as shown in Figure 5. The driving signals, which generated from a power PMAC control card (Delta Tau Data Systems, Inc., Chatsworth, CA, USA), were imposed on the three piezoelectric actuators to obtain the three-dimensional elliptical motion trajectory on tool holder. The apparatus of non-resonant 3D-EVC was fixed on the guides of the machine tools, two piezoelectric actuators (40VS12, COREMORROW, Shanghai, China) whose maximum stroke is 36 μm were placed parallel on this developed apparatus, and another one was vertically mounted. The probes of the displacement sensor were installed parallel with the piezoelectric stacks and the vibration displacement of the tool point in three directions was measured online by using a four-channel capacitive displacement sensor (Microsense DE-5300, MicroSense LLC, Lowell, MA, USA). The driving signal was amplified by a power amplifier (PI E-500, Physik Instrumente (PI) GmbH & Co. KG, Karlsruhe, Germany) with a criterion amplification factor of 1060.1.

In the experiments process, the connection between the power PMAC controller, the displacement sensor, the piezoelectric stacks and the power amplifier of the piezoelectric stack were set as a closed loop. The driving signals generated with the power PMAC controller was amplified by a power amplifier to impose on the piezoelectric stacks, and the tool holder would form the desired trajectory.

The frequency *f* and amplitude *A* of vibration was set as the process of synthetizing elliptical trajectory above. The cutting methods (i.e., TCC, 2D-EVC and non-resonant 3D-EVC) could be converted through the adjustment of the driving signal. For example, the TCC method could be achieved when the driving single in three directions was set to zero. In 2D-EVC, the phases in Formula (1) were set to φ_1_ = φ_2_ = π/2 and φ_3_ = π, respectively. Therefore, the maximum tool vibration speed in 2D-EVC was calculated as *v*_t−2D_ (max) = 2π*f*_2D_*A* = 132 mm/min, where *f*_2D_ = ω_1_ = ω_2_ = ω_3_ = 50 Hz, *A* = 7 μm. In addition, the setting of driving signals parameters in non-resonant 3D-EVC was same as the synthetic three-dimensional elliptical motion trajectory of the tool tip above. However, there was existed a certain angle γ between the cutting direction and the movement direction of the tool on the workpiece during the effective cutting stage, so the maximum tool vibration speed in non-resonant 3D-EVC was satisfied as *v*_t−3D_ (max) = 2π*f*_3D_*A* × cos^2^γ = 95 mm/min, where *f*_3D_ = ω_1_ = ω_2_ = ω_3_ = 50 Hz, *A* = 7 μm, γ = 45°.

Table 1 shows the conditions of cutting experiment where the cutting speed value was less than the maximum tool vibration speed to achieve the complete two-dimensional or three-dimensional elliptical motion trajectory on the tool tip. In order to contrast the performance of processing Ti-6A1-4V alloy using different cutting methods, the value of depth of cutting and feed rate were fixed and compensated the hysteresis error (1 μm and 0.2 mm/min), and the cutting speed was set to 30 mm/min–70 mm/min, respectively. The workpiece was Ti-6A1-4V with a diameter of 12.7 mm and a length of 50 mm, installed in the spindle of machine tools to execute the cutting experiments.

## 4. Results and Discussion

### 4.1. Cutting Marks

In order to verify the machining feasibility of the apparatus of non-resonant 3D-EVC during the processing of Ti-6Al-4V alloy, the surface cutting marks generated by the three different cutting methods (e.g., TCC, 2D-EVC and the non-resonant 3D-EVC) were discussed in detail. The cutting speed in three different cutting methods were set as 70 mm/min, the vibration frequency of the tool tip in 2D-EVC and non-resonant 3D-EVC was 50 Hz.

**Figure 6:**
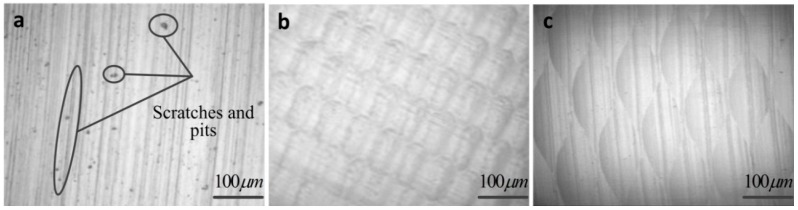
Surface topography during different processing method. (*S* = 70 mm/min, *A* = 7 μm, *D* = 1 μm and *F* = 0.2 mm·min^−1^) (**a**) Traditional continuous cutting (TCC) method; (**b**) Two-dimensional elliptical vibration cutting (2D-EVC) method with the vibration frequency of the tool tip *f*_2D_ = 50 Hz; (**c**) The non-resonant three-dimensional elliptical vibration cutting (3D-EVC) method with the vibration frequency of the tool tip *f*_3D_ = 50 Hz.

Figure 6 illustrates the surface cutting marks of machining Ti-6Al-4V alloy generated by the three different cutting methods, which are measured by a high-power microscope (CDM-500AE, Changfang Optical Instrument Co. Ltd., Shanghai, China). The surface produced by the TCC method is shown in Figure 6a. It is obvious that the peaks and valleys generated by the diamond cutter and the scratches and pits which may be caused by the formation of built-up edge on diamond cutter during the TCC process. In contrast, there are no obvious peaks and valleys in the machined surface machined by the 2D-EVC and non-resonant 3D-EVC methods, as shown in Figure 6b,c. Particularly in the process of the non-resonant 3D-EVC, the characteristic of intermittent cutting of tools reduces cutting force and prolongs tool life significantly. Simultaneously, the phenomenon of peaks and valleys are reduced considerably due to a certain angle between the movement direction of the tool on the workpiece and the direction of cutting, making it more favorable for chip removal.

### 4.2. Surface Roughness

**Figure 7:**
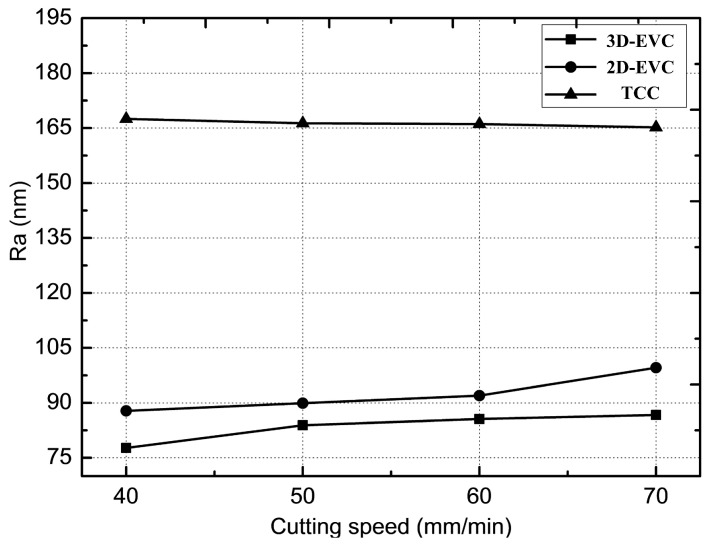
Surface roughness under different cutting speeds. (*f*_2D_ = *f*_3D_ = 50 Hz, *A* = 7 μm, *D* = 1 μm and *F* = 0.2 mm·min^−1^).

The surface roughness machined by three cutting methods was measured using the laser interferometer (NewView ZYGO, Zygo Corporation, Berwyn, PA, USA), and the surface roughness Ra were obtained by calculating the average values during 10 measurements under different cutting speeds. Figure 7 presents the surface roughness values generated three different cutting methods at four cutting speeds (e.g., 40 mm/min, 50 mm/min, 60 mm/min and 70 mm/min). In the experiments of surface roughness measurement, the cutting experiments under different cutting speeds were carried out 10 times in each cutting method (TCC, 3D-EVC and non-resonant 3D-EVC). The value of surface roughness was confirmed by the average of the 10 measurements. The distance form measuring points on a workpiece to the center of workpiece was 3 mm, and the measurement area was 420 × 420 (μm^2^).

As shown in Figure 7, the value of surface roughness obtained by the 2D-EVC and the non-resonant 3D-EVC are low relatively in comparison with the TCC method. This is because the cutting force and the wear of the tool can be significantly reduced in the cutting process due to the intermittent separation of the tool and the workpiece, and the quality of the surface could be improved simultaneously [19,33]. In addition, the value of surface roughness increases slightly with the increase of cutting speed in 2D-EVC and non-resonant 3D-EVC; this may be attributed to the slight chatter phenomenon in the tool caused by the intermittent contact between the tool and the workpiece, and the residual height of cutting surface will be changed with variation in the cutting speed. On the other hand, the time of the effective cutting stage is shortened and the length of contact between the tool and the workpiece becomes longer with the increase in the cutting speed. The impact on the tool is bigger in each cutting cycle, which will also increase the chatter phenomenon, and then increase the value of Ra. In contrast, the surface roughness machined by the TCC method is reduced slightly due to the high cutting speed could improve the cutting quality to a certain extent. The minimum roughness value can be reached 77.3 nm under a cutting speed of 40 mm/min in the non-resonant 3D-EVC process. The reason for the much smaller value of Ra is that there is a certain angle between the chip flow and the cutting direction, which reduces the wear of rake face. At the same time, there is a corresponding cutting force component in the direction of the chip flow, which further reduces the cutting force in the cutting process.

### 4.3. Different Surface Topography in Ti-6Al-4V Alloy with Varies Cutting Speed and Vibration Frequency

In general, the microscopic surface topography is an important indicator to verify the cutting machinability. The effects of cutting speed (30 mm/min, 40 mm/min, 50 mm/min, 60 mm/min, and 70 mm/min) on surface topography in processing of the non-resonant 3D-EVC were experimentally investigated. The frequency of vibration was setting as 50 Hz.

**Figure 8:**
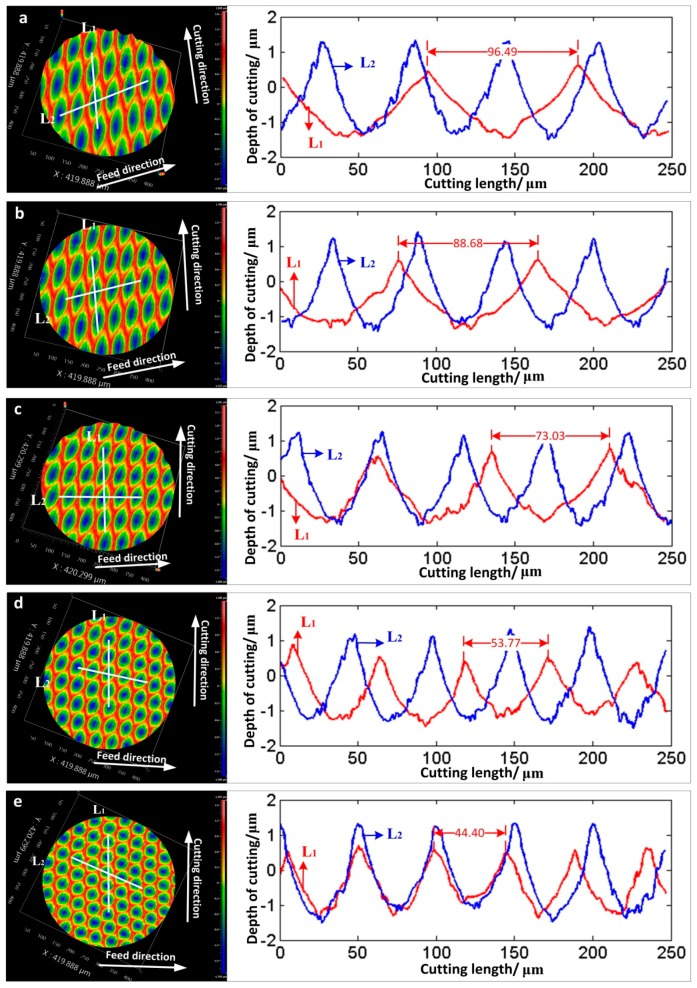
Effect of cutting speed on surface topography machined by a non-resonant three-dimensional elliptical vibration cutting (3D-EVC) apparatus and the section contours along the cutting direction (Red line L_1_) and feed direction (Blue line L_2_), respectively (*f*_3D_ = 50 Hz, *A* = 7 μm, *D* = 1 μm and *F* = 0.2 mm·min^−1^). (**a**) *S* = 70 mm/min; (**b**) *S* = 60 mm/min; (**c**) *S* = 50 mm/min; (**d**) *S* = 40 mm/min; (**e**) *S* = 30 mm/min.

Figure 8 represents the effects of cutting speed on surface topography and the section contours along the cutting direction (red line L_1_) and feed direction (blue line L_2_), respectively. As shown in Figure 8, the dimension of the elliptical cutting mark in machined surface is decreased with the increase of the cutting speed. In particular, it is obvious that the length of long axis which has a certain angle with cutting direction in single elliptical cutting mark decreases significantly. The length of long axis is almost same as the short axis when the cutting speed was reduced to 30 mm/min, and the shape of cutting marks can be approximately seen as circular. For the section contour along the cutting direction, it could be shown that there is a certain distance deviation between two adjacent ellipses on machined surface under different cutting speeds. More ellipses topography in the cutting mark will be obtained with the decrease of the cutting speed. The maximum distance deviation was 28.63 μm with the cutting speeds 70 mm/min and 30 mm/min. However, there are not obvious changes between two adjacent ellipses on the machined surface along the feed direction. Therefore, the shape and dimension of elliptical cutting marks are related to the cutting speed, while the different cutting speed would not affect the interval of two adjacent ellipses on machined surface.

**Figure 9:**
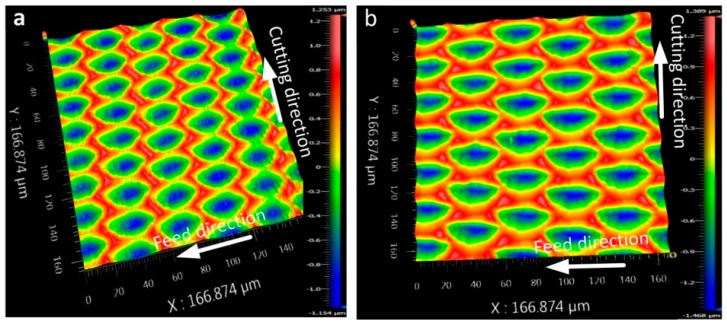
Effect of vibration frequency on surface topography machined by non-resonant three-dimensional elliptical vibration cutting (3D-EVC) apparatus (*S* = 50 mm/min, *A* = 7 μm, *D* = 1 μm and *F* = 0.2 mm·min^−1^). (**a**) The vibration frequency in the tool tip was *f*_3D_ = 50 Hz; (**b**) The vibration frequency in the tool tip was *f*_3D_ = 30 Hz.

In order to explain the reason for the variety of intervals between two adjacent elliptical cutting marks, comparison experiments with different vibration frequency (30 Hz and 50 Hz, respectively) have been carried out using the non-resonant 3D-EVC method. The maximum tool vibration speed in non-resonant 3D-EVC, corresponding to 30 Hz, was calculated as 57.2 mm/min. Therefore, the cutting speed in this process was set as 50 mm/min. The surface topography obtained under these conditions is shown in Figure 9. As shown in Figure 9, the interval of each elliptical cutting mark along the cutting direction is decreased due to the augment of the vibration frequency. In other words, the effect of residual cutting area remained by the last cutting cycle may be attributed to the increase in the surface roughness under the lower vibration frequency.

### 4.4. Machinability Analysis of Freeform Surface in Non-Resonant 3D-EVC

With the wide application of titanium alloy in different fields, the machining of freeform surface and special functional surface has begun to appear in the field of ultra-precision. In order to validate the feasibility of freeform surface on difficult-to-cut materials based on the non-resonant 3D-EVC apparatus, the freeform surface of Ti-6Al-4V alloy is machined by this developed apparatus in this paper.

**Figure 10:**
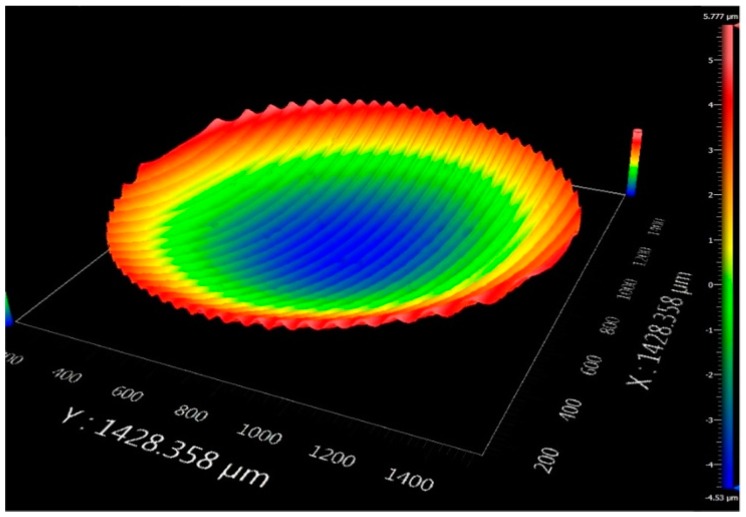
Concave spherical surface topography of titanium alloy machined by a non-resonant three-dimensional elliptical vibration cutting (3D-EVC) apparatus. (*f*_3D_ = 50 mm/min, *S* = 50 mm/min, *A* = 7 μm, *D* = 1 μm and *F* = 0.2 mm·min^−1^).

**Figure 11:**
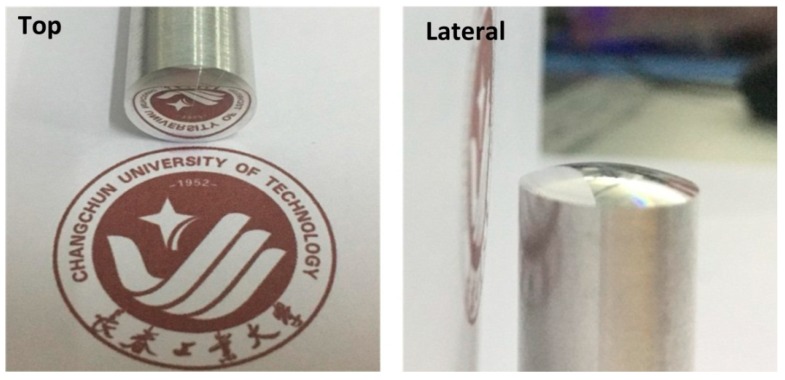
Convex spherical surface topography of titanium alloy machined by a non-resonant three-dimensional elliptical vibration cutting (3D-EVC) apparatus (*f*_3D_ = 50 mm/min, *S* = 50 mm/min, *A* = 7 μm, *D* = 1 μm and *F* = 0.2 mm·min^−1^). (**left**) Top view; (**right**) Lateral view.

In order to obtain the better freeform surface and reduce tool wear during the machining of Ti-6Al-4V alloy, the cutting method of multiple cuttings and step-by-step chip removal was adopted in the processing of freeform surface. The vibration frequency of the tool tip and the cutting speed were set as 50 Hz and 50 mm/min, respectively. The concave spherical surface topography of Ti-6Al-4V alloy was machined using the non-resonant 3D-EVC, as shown in Figure 10. As shown in Figure 10, the center of Ti-6Al-4V alloy was descended gradually and smoothly with the successive feed of the cutting lay, which proved the machining feasibility of the freeform surface. In addition, the convex spherical surface topography of Ti-6Al-4V alloy machined using this developed apparatus also has a great mirror quality, as shown in Figure 11, which proved that the machinability of freeform surface by non-resonant 3D-EVC technique in Ti-6Al-4V alloy.

## 5. Conclusions

The cutting experiments research on Ti-6Al-4V alloy was carried out by using the apparatus of non-resonant three-dimensional elliptical vibration cutting (3D-EVC). The principle of non-resonant 3D-EVC and the model of cutter motion were introduced, and then the tool path of the developed apparatus was synthesized. Based on the theoretical analysis, the comparison experiments were carried out to process Ti-6Al-4V alloy by the TCC, 2D-EVC and non-resonant 3D-EVC methods. Finally, the influence of the cutting speed, the vibration frequency on the surface topography and the machinability of the freeform surface of the titanium alloy were studied. The conclusions can be drawn as follows:

1. The non-resonant 3D-EVC technique can significantly improve the surface integrity of titanium alloys, and reduce the scratches and pits. More alterable cutting marks and more excellent quality could be obtained compared with the TCC and 2D-EVC methods. The minimum roughness value can be reached 77.3 nm under a cutting speed of 40 mm/min.
2. In the non-resonant 3D-EVC process, the dimension of long axis, which has a certain angle with cutting direction in a single elliptical cutting mark, decreases significantly. The length of the long axis is almost same as the short axis when the cutting speed was reduced to 30 mm/min, and the shape of cutting marks can be approximately seen as circular. The spacing of adjacent elliptical cutting marks along the cutting speed direction depends on the vibration frequency.
3. The concave/convex spherical surface topography are achieved, which proved the machinability of the freeform surface by the non-resonant 3D-EVC technique in Ti-6Al-4V alloy.

In the future, the research of improving closed-loop control accuracy and the higher vibration frequency of the tool tip in non-resonant 3D-EVC apparatus will be studied.

## Figures and Tables

**Table 1:** The specific experimental conditions.

Type	Parameter	Value
Workpiece	Material	Ti-6A1-4V
	Length (mm)	50
	Diameter (mm)	12.7
Diamond tool	Nose radius (mm)	0.2
	Rake angle (°)	0
	Clearance angle (°)	7
Cutting parameter	Depth of cutting (*D*/μm)	1
	Feed rate (*F*/mm·min^−1^)	0.2
	Cutting speed (*S*/mm·min^−1^)	30–70
	Coolant type	Dry cutting
Vibration	Frequency (*f*/Hz)	30, 50
	Amplitude (*A*/μm)	7

## Nomenclature

*t_i_* (*i* = 0, 1 , 2, 3)	Time point of cutting process
*h*	The maximum depth of cut (μm)
α	Rotation angle of Plane 1 (°)
β	Rotation angle of Plane 2 (°)
γ	The angle between the movement direction of tool on workpiece and the cutting direction (°)
*P_i_* (*i* = 1, 2, 3)	Position point of cutting process
*z*, *y*_1_, *y*_2_	The ideal vibration displacement in *Z*, *Y*1 and *Y*2 directions of tool tip, respectively (μm)
*A*_1_, *A*_2_, *A*_3_	Amplitudes of driving singles in *Z*, *Y*1 and *Y*2 directions, respectively (μm)
ω_1_, ω_2_, ω_3_	Angular frequency in *Z*, *Y*1 and *Y*2 directions, respectively (Hz)
φ_1_, φ_2_, φ_3_	Phase in the driving signals along *Z*, *Y*1 and *Y*2 directions, respectively
*t*	Time (s)
*z*’, *y*_1_’, *y*_2_’	The real displacement of three piezoelectric stacks (μm)
θ	The phase difference between two piezoelectric stacks installed parallel on hinge platform (°)
*l*_1_, *l*_2_	The vertical distance from the driving point implemented on the flexible hinge along the *Y*1 and *Y*2 directions to the plane paralleled to the flexible hinge along the *Z* direction of diamond tool, respectively (mm)
*l*	The linear distance from tool tip point to the plane of the two piezoelectric stacks in *Y*1 and *Y*2 directions (mm)
*x*_t_, *y*_t_, *z*_t_	Spatial coordinate system
*f*_2D_, *f*_3D_	Vibration frequency in 2D-EVC and non-resonant 3D-EVC methods, respectively
*D*, *S*, *F*	Depth of cutting, cutting speed and feed rate, respectively
*A*	Vibration amplitude
